# A Modified Empirical Wavelet Transform for Acoustic Emission Signal Decomposition in Structural Health Monitoring

**DOI:** 10.3390/s18051645

**Published:** 2018-05-21

**Authors:** Shaopeng Dong, Mei Yuan, Qiusheng Wang, Zhiling Liang

**Affiliations:** 1 School of Automation Science and Electrical Engineering, Beihang University, Beijing 100191, China; dspsx@buaa.edu.cn (S.D.); wangqiusheng@buaa.edu.cn (Q.W.); zhiling310@buaa.edu.cn (Z.L.); 2 Collaborative Innovation Center of Advanced Aero-Engine, Beihang University, Beijing 100191, China

**Keywords:** structural health monitoring, acoustic emission, empirical wavelet transform

## Abstract

The acoustic emission (AE) method is useful for structural health monitoring (SHM) of composite structures due to its high sensitivity and real-time capability. The main challenge, however, is how to classify the AE data into different failure mechanisms because the detected signals are affected by various factors. Empirical wavelet transform (EWT) is a solution for analyzing the multi-component signals and has been used to process the AE data. In order to solve the spectrum separation problem of the AE signals, this paper proposes a novel modified separation method based on local window maxima (LWM) algorithm. It searches the local maxima of the Fourier spectrum in a proper window, and automatically determines the boundaries of spectrum segmentations, which helps to eliminate the impact of noise interference or frequency dispersion in the detected signal and obtain the meaningful empirical modes that are more related to the damage characteristics. Additionally, both simulation signal and AE signal from the composite structures are used to verify the effectiveness of the proposed method. Finally, the experimental results indicate that the proposed method performs better than the original EWT method in identifying different damage mechanisms of composite structures.

## 1. Introduction

Composite materials are widely used in aircraft structures because of their advantages of stiffness, strength, light weight and excellent fatigue and corrosion resistance [1,2,3]. However, the various damage mechanisms of composite structures are very difficult to predict. Structural health monitoring (SHM), which can assess the structure status in real time and supply an early warning, has been proposed to confront this challenge [4,5]. Acoustic emission (AE) is an attractive technique for SHM which is sensitive, can detect various types of composite material damage and needs only a small number of sensors [3,6,7]. The exact health state can be obtained by monitoring the AE signals. Furthermore, damage information such as damage mechanisms, damage position and residual life could also be extracted when the AE signals can be accurately collected and analyzed [8]. At present, both traditional AE characteristic parameters and new signal processing methods can be used to analyze AE signals.

When composite structures are exposed to an external load, AE signals will occur from matrix cracking, matrix/fiber debonding, delamination, and fiber fracture [9]. Following the theory of plate wave, the generated waves will propagate in all directions, and form different modes. The difference of these modes is due to the particle displacement: in the plane (IP) for the symmetric or extensional mode and outside to the plane (OP) for the antisymmetric or flexural mode [10]. For the extensional mode, all frequency components travel at the same velocity, but for the flexural mode, the high frequency components travel faster than the low frequency components [11]. In practical structures, the signals detected by AE sensors depend on various factors, such as the structures’ properties (including the material, width, geometry, and so on), damage source orientation, damage location, signal attenuation, wave dispersion, boundary reflections and other interference phenomenon. Therefore, all these factors have to be taken into account during the AE data analysis [10].

A major issue is how to distinguish between the different damage mechanisms according to the characteristics of the AE signal. Many studies have used time features, frequency characteristics, or time-frequency methods to identify the damage mechanisms [10,11,12,13,14], but they were only applied in simple laboratory experiments. For complex structures, Sause and Hamstad established an effective finite element modeling (FEM) routine to interpret the AE signal behavior [15,16,17,18,19,20], which can give advice about how to extend the laboratory results to a practical scenario. In order to assess the real-time damage mechanisms, Martínez-Jequier et al. [21] designed appropriate hardware frequency filters to split the extensional and flexural modes of the damage AE signals; Yaacoubi and Dahmene [22] thought about the large data sets of the collected data during the composite SHM, and explored various innovative processing techniques to save computing time. Additionally, based on the variety of signal processing methods, a large number of studies have correlated specific AE signal features with the progression of damage. Gutkin et al. [23] identified some damage mechanisms of CFRP through pattern recognition algorithm based on five representative AE parameters (peak amplitude, peak frequency, energy, rise time and duration). McCrory et al. [24] classified the damage of matrix and delamination of CFRP based on AE signals through three classification techniques. Kumar et al. [25] considered significant AE parameters, such as counts, energy, signal strength, absolute energy and hits, and used an artificial neural network to predict the failure strength of composite laminates. Beheshtizaeh and Mostafapour [26] indicated that wavelet analysis was suitable to process the AE signals in three-point bending load of composites structure, but the proper wavelet type should be selected.

Recently, inspired by wavelet transform and empirical mode decomposition (EMD) [27], the empirical wavelet transform (EWT) has been developed by Gilles [28,29]. Amezquita-Sánchez et al. [30] employed EWT to estimate both natural frequencies and damping ratios of large civil structures. Yuan and Sadhu [31] used EWT to separate the closely-spaced natural frequencies of structures. The main idea of EWT is to determine the Fourier segments and then design a series of wavelet filters to decompose the signal into several sub-signals. EWT became adaptive when the Fourier segments were automatically determined. The empirical rule was to find all the local maxima of the spectrum and then use the center of the two adjacent local maxima as the boundaries of Fourier segments [32]. Some researchers have proposed solutions to modify the Fourier segments. Zheng et al. [33] proposed a method of adaptive parameterless EWT to fulfill an adaptive separation of the Fourier spectrum. Wang et al. [34] developed the sparsity-guided EWT to automatically establish Fourier segments. Song et al. [35] employed the scale-space histogram segmentation to determine the boundaries adaptively. However, for damage AE signals, when strong noise or frequency dispersion exist in the Fourier spectrum of the signals, such Fourier segmentation solutions are not always reliable and useful.

In this paper, local window maxima (LWM) algorithm is proposed to automatically determine the boundaries of Fourier segments, and it is appropriate for EWT to analyze the damage AE signals of composite structures. The key ideas of the algorithm are to search all the local maxima of the Fourier spectrum in an optimal window and then to obtain the segmentation boundaries. Since different damage mechanisms for composite structures have different frequency ranges, separating the different damage mechanisms is equivalent to decomposing the AE signals in the Fourier spectrum. The analysis results on a simulation signal show that this method can effectively obtain the peak frequency bands. In addition, the pencil-lead breaks (PLB) and three-point bending experiments on the composite structure is carried out to prove the applicability of the proposed solution.

## 2. The Modified Empirical Wavelet Transform Method

**Figure 1:**
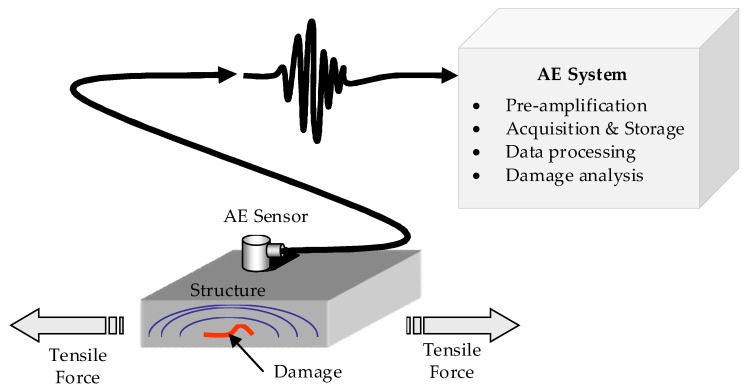
The structure diagram of AE detection theory.

The structure diagram of AE detection theory is shown in Figure 1. When any damage occurs in the structure under the action of external forces, a transient wave which is the result of the sudden release of stored energy, propagates from the damage location and eventually reaches the surface of the structure. The surface displacement can be converted into an electrical signal by an AE sensor, and the sensor signal can be pre-amplified, acquired, processed and analyzed by the AE system.

### 2.1. The EWT Method

As shown in Figure 1, data processing is an important procedure in the AE system. The aim of EWT method was to extract the signal modes, therefore it could be applied to process the AE signals. The EWT process contains three important aspects: (1) in segmenting the spectrum of the signal, the first step was to detect all the local maxima of the spectrum, then get the boundaries which were the midpoint of two consecutive maxima, using the algorithm given in Equation (1); (2) constructing the empirical wavelets by defining a bank of wavelet filters composed of one low-pass filter and *N* − 1 band filters based on the boundaries, which is given in Equations (2)–(5); (3) extracting the empirical modes by applying the wavelet filter banks to divide the signal into frequency sub-bands. More detailed information can be found in reference [28].

(1) $$w_{n}=\{\begin{array}{ll} 0, & n=0\\ \frac{\Omega_{n}+\Omega_{n+1}}{2}, & n=1,2,\ldots ,N-1\\ \pi , & n=N \end{array},$$

where $\omega_{n}$ is the segmentation boundaries; $\Omega_{n}$ is the corresponding angular frequency of the local maxima.

(2) $${\hat{\psi }}_{n}(\omega )=\{\begin{array}{ll} 1, & (1+\gamma )\omega_{n}\leq \left|\omega \right|\leq (1-\gamma )\omega_{n+1}\\ \cos\left[\frac{\pi }{2}\beta \left(\frac{1}{2\gamma \omega_{n+1}}(\left|\omega \right|-(1-\gamma )\omega_{n+1})\right)\right], & (1-\gamma )\omega_{n+1}\leq \left|\omega \right|\leq (1+\gamma )\omega_{n+1}\\ \sin\left[\frac{\pi }{2}\beta \left(\frac{1}{2\gamma \omega_{n}}(\left|\omega \right|-(1-\gamma )\omega_{n})\right)\right], & (1-\gamma )\omega_{n}\leq \left|\omega \right|\leq (1+\gamma )\omega_{n}\\ 0, & otherwise \end{array},$$

(3) $${\hat{\phi }}_{n}(\omega )=\{\begin{array}{ll} 1, & \left|\omega \right|\leq (1-\gamma )\omega_{n}\\ \cos\left[\frac{\pi }{2}\beta \left(\frac{1}{2\gamma \omega_{n}}(\left|\omega \right|-(1-\gamma )\omega_{n})\right)\right], & (1-\gamma )\omega_{n}\leq \left|\omega \right|\leq (1+\gamma )\omega_{n}\\ 0, & otherwise \end{array},$$

where ${\hat{\psi }}_{n}(\omega )$ is the empirical wavelets, ${\hat{\phi }}_{n}(\omega )$ is the empirical scaling function; γ ensures no overlap between two consecutive segments, β(x) is an arbitrary function, they are given as follows:

(4) $$\gamma <\min_{n}\left(\frac{\omega_{n+1}-\omega_{n}}{\omega_{n+1}+\omega_{n}}\right)\;$$

(5) $$\beta (x)=\{\begin{array}{ll} x^{4}(35-84x+70x^{2}-20x^{3}), & 0<x<1\\ 0, & x\leq 0\\ 1, & x\geq 1 \end{array}$$

### 2.2. The LWM-EWT Method

The process of spectrum segmentation is the basis and is very important for the EWT algorithm to achieve ideal separation results. When the signal has a relatively obvious peak frequency, the above-mentioned segmentation method is effective. However, when AE signals from the damage characteristics that have some frequency bands are processed, some local maxima which are from noise or frequency dispersion might appear and be mistakenly kept in the peak frequency sequence, then this method may lead to a false segmentation. Another drawback of the spectrum segmentation is that the boundary method, which is to obtain the midpoint of the two adjacent local maxima, is too simple, especially when the distance of two local maxima is far, and the extracted signal bandwidth will be too wide.

When the EWT method is used to process the AE signals, a modified method is proposed in this paper. This method can avoid the improper segments, detect the local maxima and obtain the frequency boundaries. The method is the local window maxima (LWM) segmentation algorithm which means finding the local maxima in a proper window which can be determined according the damage signal features. The process is as follows:

1. Use the FFT algorithm to obtain the spectrum of the AE sensor signal.
2. According to the characteristics of the AE signals from composite structures, set the window value *W* and the segmentation numbers *N* (the two parameters are discussed in Section 3).
3. Set the counts of local window maxima *m* = 0.
4. Search all the local maxima of the signal spectrum.
5. Sort descending the local maxima, save the first one as one LWM and set m=m+1.
6. Center on the LWM which is generated from step 5 and zero other spectrum values in W.
7. If m is not equal N, go back to step 4.
8. Calculate the minimum distance *L* of all the two consecutive LWM.
9. Obtain the boundaries according to the *L* and each LWM, for example, if LWM-1 is the first LWM and $\Omega_{1}$ is the frequency of LWM-1, then boundaries for LWM1 are given in Equation (6):
  (6) {ω0=Ω1−L2ω1=Ω1+L2

**Figure 2:**
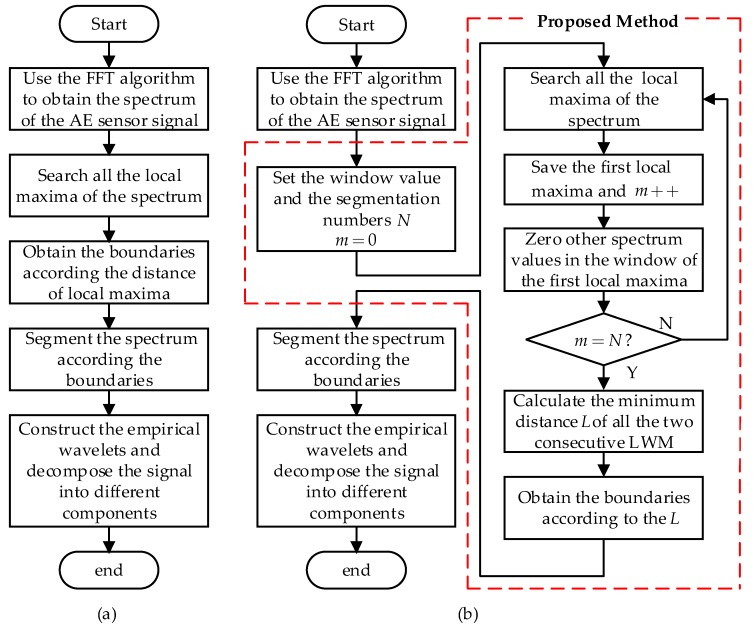
Flowcharts of (**a**) the EWT method and (**b**) the proposed LWM-EWT method.

The procedure of the LWM-EWT method is compared with the EWT method in Figure 2 and the improvements are highlighted in the dotted red area.

The purpose of the proposed method is to find the proper local maxima and how to obtain boundaries. Here the details are discussed. To detect the LWM, the idea is that the most important maxima in the magnitude of the Fourier transform of the sensor signal is significantly larger than the other existing maxima.

#### 2.2.1. Picking Out the LWM

Here the example how to pick out the first LWM is given to explain the detection theory of LWM. First, the detected local maxima in the magnitude of the Fourier spectrum is sorted descending and the set can be described as:

(7) $${\left\{M_{k}\right\}}_{k=1}^{N},\;M_{1}\geq M_{2}\geq \ldots \geq M_{N},$$

where $M_{k}$ is one of the local maxima.

The corresponding angular frequencies of these maxima are given as:

(8) $$\Omega ={\left\{\Omega_{k}\right\}}_{k=1,2,\ldots ,N},$$

where $\Omega_{k}$ is the angular frequency of $M_{k}$.

If $M_{1}$ is the first LWM, denoted by LWM-1, then zero the amplitude that the frequency is in the window of W, except for the center frequency of $\Omega_{1}$. The equation is as follows:

(9) $$\left|X(\omega )\right|=0,\;\Omega_{1}-\frac{W}{2}\leq \omega \leq \Omega_{1}+\frac{W}{2},\;\omega \neq \Omega_{1},$$

where |X(ω)| is the spectrum amplitude; ω is the angular frequency.

**Figure 3:**
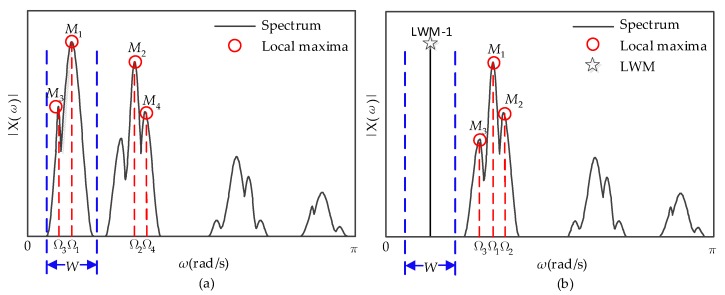
The detection process of the first LWM. (**a**) Detect the four local maxima and determine the window. (Red circles are the local maxima; blue dashed lines are the boundaries of the window.); (**b**) zero the amplitude in the window, obtain the first LWM which is denoted by pentagram, and redetect the other three local maxima which are denoted by the red circles.

When there are four LWM that need to be detected, the detection process of the first LWM is illustrated in Figure 3.

#### 2.2.2. Detecting the Boundaries

All the LWM can be obtained based on the solution in Section 2.2.1, then the minimum distance of all the two adjacent LWM can be calculated by the following equation:

(10) $$L=\min(\Omega_{i+1}-\Omega_{i})\;i=1,2,3,\ldots ,N$$

**Figure 4:**
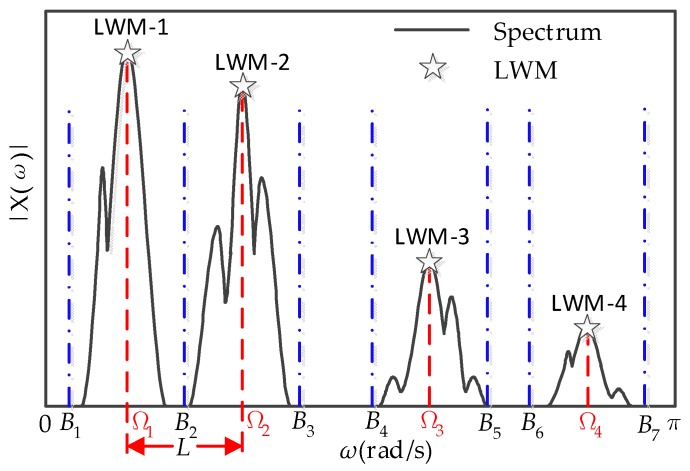
The detected boundaries which are denoted by the blue dot-dashed lines, the four LWM and the minimum distance of two adjacent LWM.

And the boundaries are given as:

(11) Bj={Ωi−L2Ωi+L2     i=1,2,…,N; j=1,2,…,2N−1

when there are four LWM, the boundaries are shown in Figure 4.

## 3. Numerical Simulation Signal Analysis and Discussion

A numerical simulation is done to verify the proposed method. Without loss of generality, the modulated sine burst, which is a narrow-band signal, is employed in the simulation. From [36], the simulation signal can be expressed as follows:

(12) $$x(t)=A[H(t)-H(t-\frac{N}{f_{C}})][1-\cos(\frac{2\pi f_{c}t}{N})]\sin(2\pi f_{c}t),$$

where *A* is the signal amplitude; $f_{c}$ is the central frequency; t is the time; H(t) is the Heaviside step response; N is the wave numbers.

**Figure 5:**
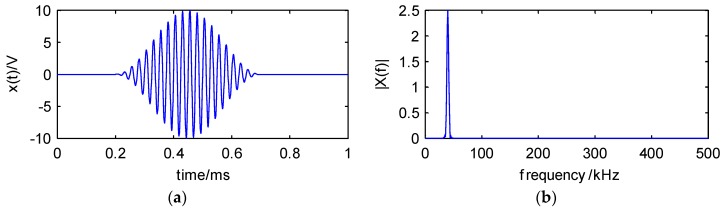
The (**a**) waveform and (**b**) spectrum of the simulation signal.

For example, when A=5, N=20, *t* = 1 ms, $f_{C}=40\;kHz,$ and the sample rate is 1 MHz, the simulation signal is shown in Figure 5.

During the simulation process, the first step is to generate the mixed signal according to the Equation (12), if it is composed of *n* different modes, then it can be expressed as:

(13) $$s(t)=\sum_{i=1}^{n}x_{i}(t)$$

**Figure 6:**
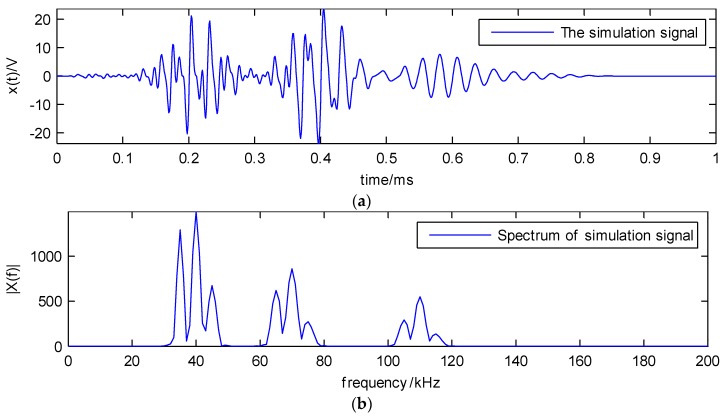
The (**a**) waveform and (**b**) spectrum of simulation signal which is generated by adding 9 different modulated sine burst signals together (the simulation parameters are in Equation (14) and the expression of each signal is in Equation (12)).

When *n* = 9, and all the simulation parameters are as given in Equation (14), the simulated sensor signal is shown in Figure 6.

(14) $$\begin{array}{l} A=\left[\begin{array}{lllllllll} 4 & 3 & 2 & 3 & 2 & 1 & 2 & 1 & 0.5 \end{array}\right]\\ N=\left[\begin{array}{lllllllll} 30 & 30 & 30 & 40 & 40 & 40 & 50 & 50 & 50 \end{array}\right]\\ f_{c}=\left[\begin{array}{lllllllll} 40 & 35 & 45 & 70 & 65 & 75 & 110 & 105 & 115 \end{array}\right] \end{array}$$

It is difficult to separate the simulated sensor signal into different components only from the waveform information in Figure 6a. But there are nine different frequency peaks in the spectrum (Figure 6b). Here the parameters of window value and segmentation numbers will be discussed. The window value relies on the practical requirement and experience, for example if the three components of 35, 40 and 45 kHz can be regarded as only one component, the window will be 10 kHz, but the segmentation numbers can be obtained by the energy of the component, if the energy proportion of one component is larger than a specify value, then the component can be considered as a segmentation.

**Figure 7:**
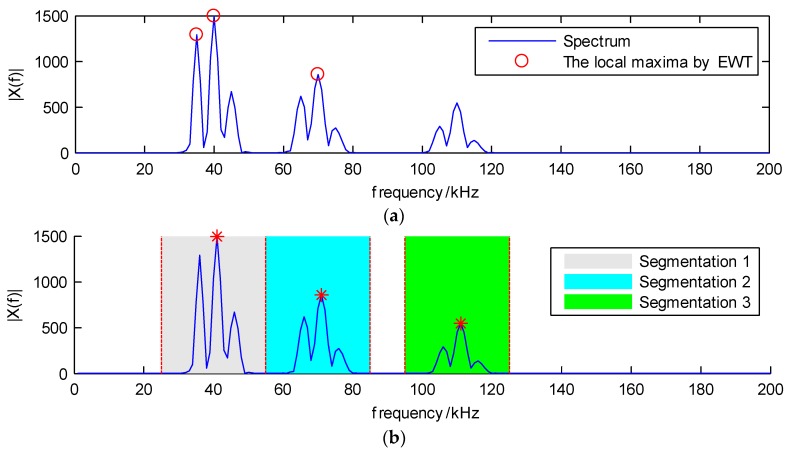
Local maxima by (**a**) the original EWT method denoted by red circles and (**b**) the LWM method denoted by red stars; the spectrum segmentation results are shown by the color of gray, cyan and green.

When the window value is 10 kHz and the segmentation numbers are three, the detected local maxima of the original EWT and the LWM method, which are the red circles and red stars, respectively, are illustrated in Figure 7. As shown in Figure 7a, the first two local maxima are very close, which may generate two narrow bands in the decomposition results, and this is not applicable for analyzing the non-stationary AE signals which have the dispersion phenomenon when they propagate on the composite structure. Therefore, compared with the original EWT method, the proposed LWM approach gives better results for the local maxima, which are shown in Figure 7b.

Based on the local maxima, segmentation boundaries are obtained which are the red dashed lines in Figure 7b, and three spectrum segmentations are shown, denoted by the gray, cyan and green colors. This indicates that the proposed LWM algorithm performs well in searching for the local maxima and segmenting the spectrum.

**Figure 8:**
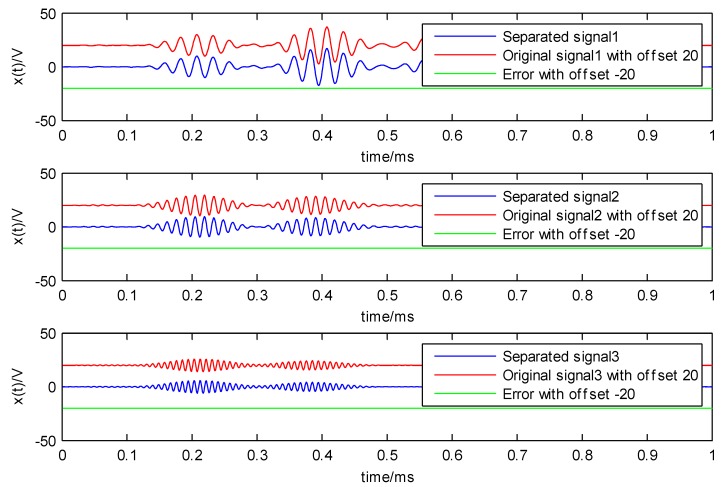
Decomposition signals of the proposed LWM-EWT method: the red curves are the original signals (with offset of 20 V); the blue curves are the separated signals and the green curves are the error (with offset of −20 V) between separated signals and original signals.

**Figure 9:**
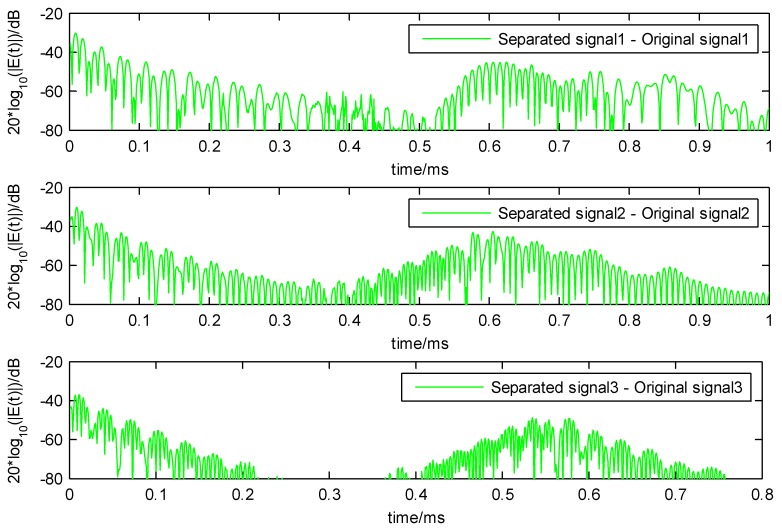
The difference value between separated signals and original signals calculated by 20*log_10(|E(t)|), the difference values are all less than 0.1 V.

The decomposition results of the LWM-EWT method are depicted in Figure 8 and Figure 9, in order to have a more obvious contrast, the original signals with offset of 20 V (each of which is the mixed signal of three nearest frequency simulation signal), the separated signals and error with offset of −20 V between them are depicted in one figure.

## 4. Experimental Results and Discussion

### 4.1. Pencil-Lead Breaks Experiment

**Figure 10:**
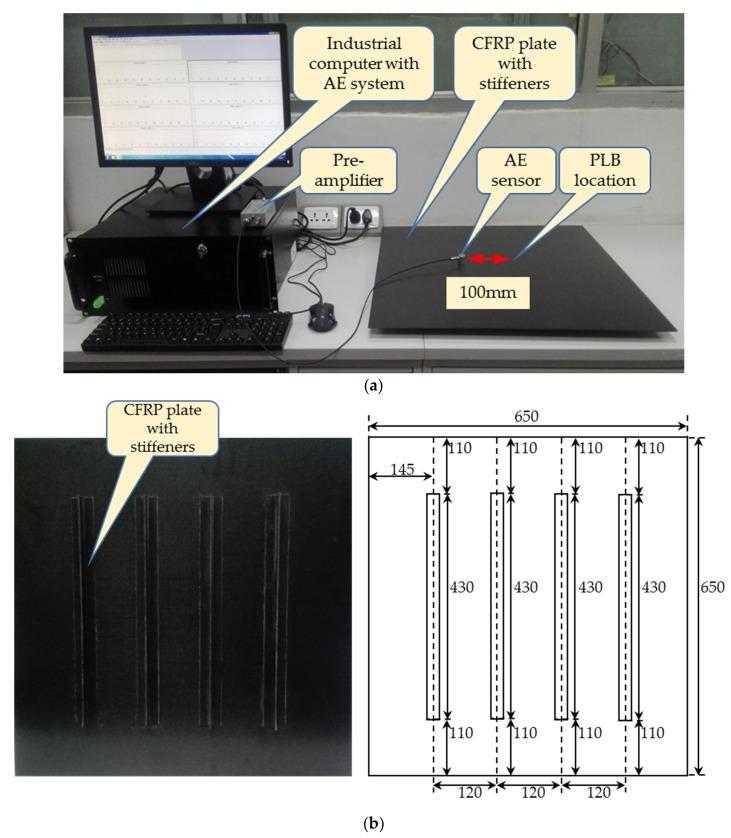
(**a**) The experimental system of PLB and (**b**) the configuration of CFRP plate with T stiffeners.

Pencil-lead breaks (PLB) in plates were used as the typical AE test source in literature [19,21]. In order to verify the effectiveness of the proposed approach, here a PLB signal is captured by an AE detection system. The schematic diagram of the experimental system is displayed in Figure 10. It consists of a CFRP plate, PLB (on the top surface of the plate), AE sensor, pre-amplifier, AE system and industrial computer. The AE sensor is mounted on the top surface of CFRP plate, and the AE signal is captured in the experiment. During the experiment, the original AE signal is amplified by 40 dB, and the sample rate is 10 MHz.

The detailed information of the experimental system is shown in Table 1.

**Figure 11:**
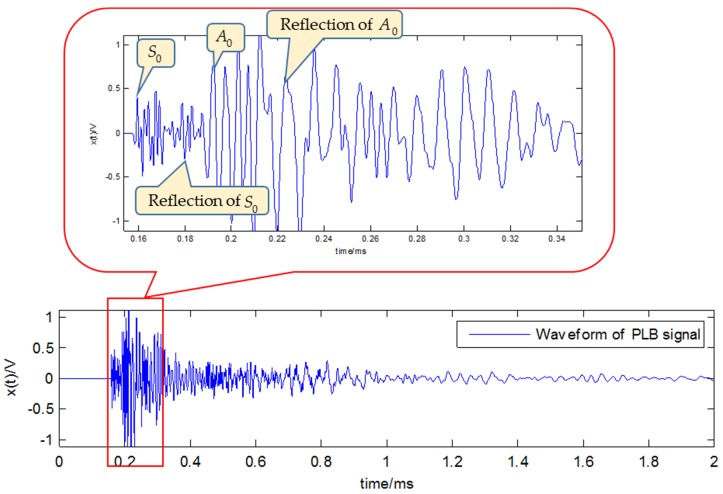
The waveform of the PLB signal.

**Figure 12:**
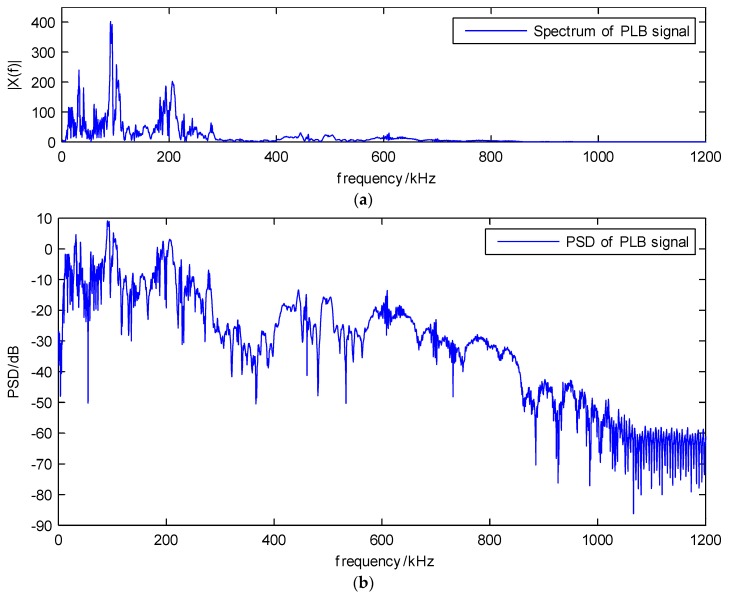
(**a**) The spectrum and (**b**) the PSD of PLB signal. (Include three different main spectrum bands, which are near the 100, 200 and 500 kHz marks).

From the acquired AE signal, the waveform and spectrum of PLB can be obtained. The waveform in Figure 11 shows the duration of PLB signal is about 0.4 ms, and it can be seen that both the extensional mode and flexural mode are present. There are many peak frequencies, due to the big difference of the amplitude, the high frequency components are not obvious in Figure 12a, but from the power spectral density (PSD) in Figure 12b, it can be seen that the three main spectrum bands are in the near zone of 100, 200 and 500 kHz. Then the main difficulty of analyzing this signal would be separating the three main components from the high level of noise, especially the nearby peak frequencies.

**Figure 13:**
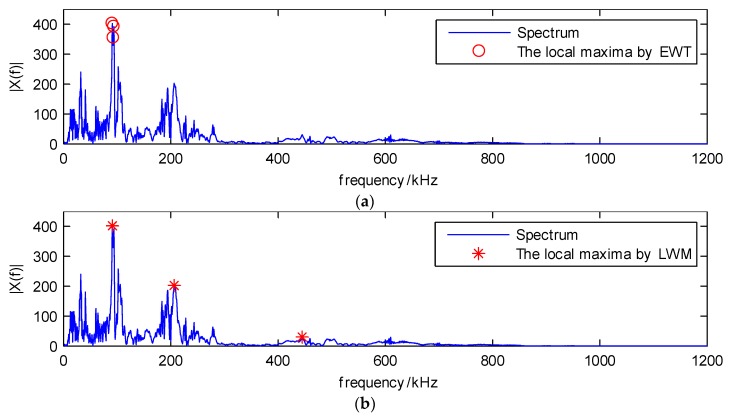
Local maxima of (**a**) the original EWT method and (**b**) the proposed LWM method.

When the window value is 50 kHz and the segmentation numbers are three, the detected local maxima using the original EWT and LWM methods are shown in Figure 13, where they are denoted by the red circles and red stars, respectively.

**Figure 14:**
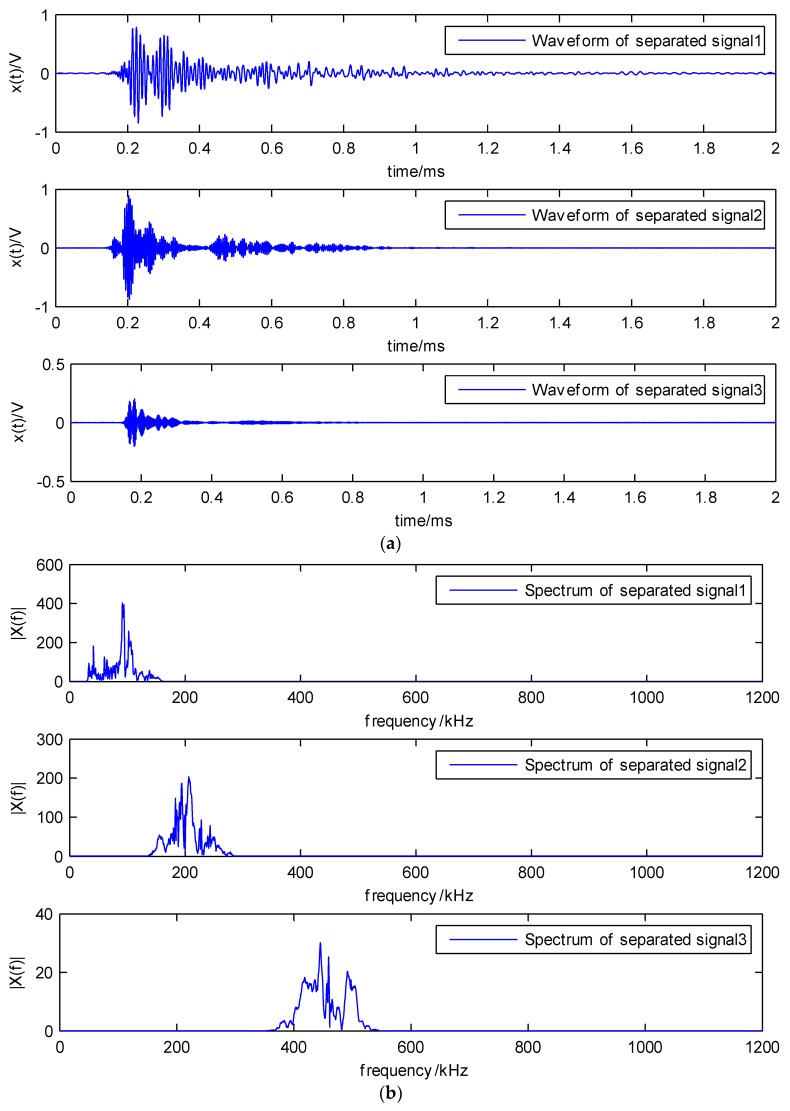
(**a**) The waveforms and (**b**) the spectrum of separated signals by the LWM-EWT method.

In Figure 13a, the three maxima are very close to each other, they should not be separated to three different components, but by using the traditional method, they could be roughly separated. Therefore, the results through the proposed LWM method are better than the original EWT, where the three local maxima are located in the three main frequency bands. The separated results based on the proposed LWM-EWT method are depicted in Figure 14. It contains the waveform and spectrum of three dominant components decomposed from the PLB signal.

### 4.2. Three-Point Bending Experiment

**Figure 15:**
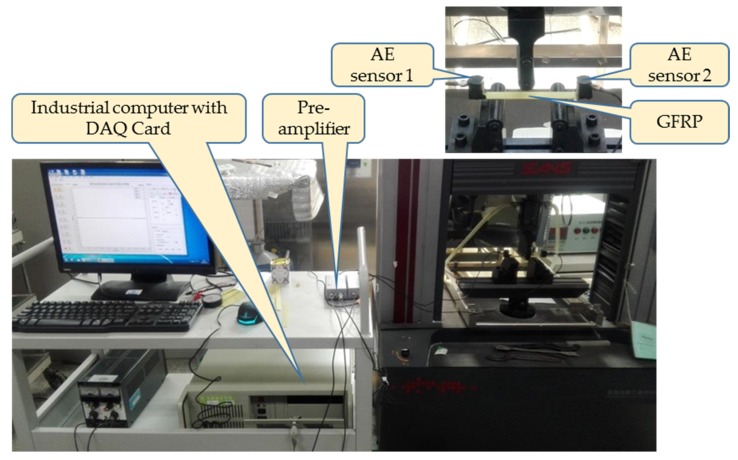
The three-point bending load experimental system.

According to the ASTM-D790-10 specification, a specimen of glass fiber reinforced plastics (GFRP) laminates plate with dimension of 120 mm × 15 mm × 2.5 mm is tested on the three-point bending load rig. The experimental system is seen in Figure 15.

**Figure 16:**
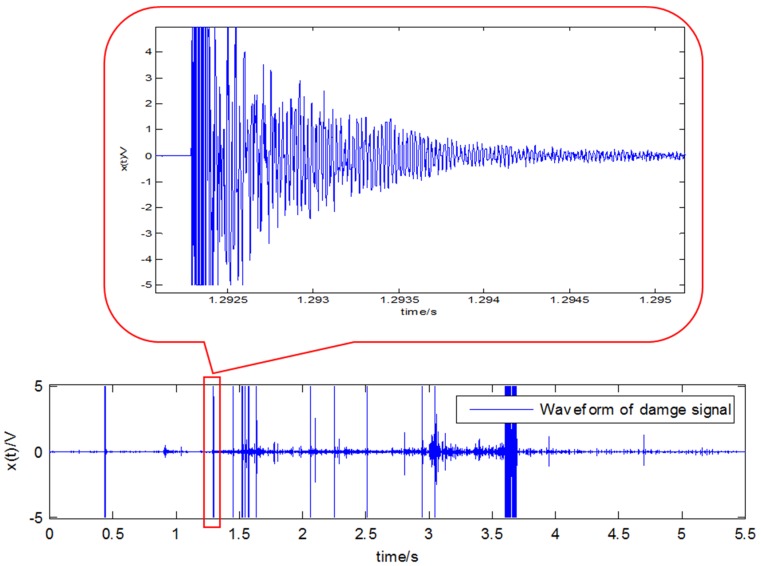
The damage signal waveform of sensor 1.

During the experimental process, which lasts about 5 s, two AE sensors are used to monitor the damage signals, and the waveform of sensor 1 is seen in Figure 16.

**Figure 17:**
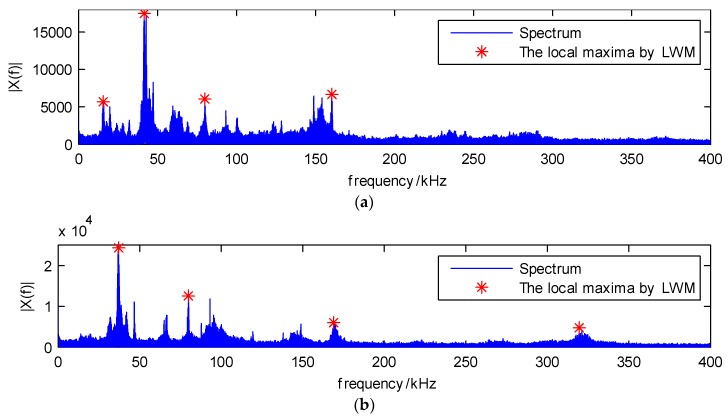
The spectrum and local maxima of (**a**) sensor 1 and (**b**) sensor 2.

**Figure 18:**
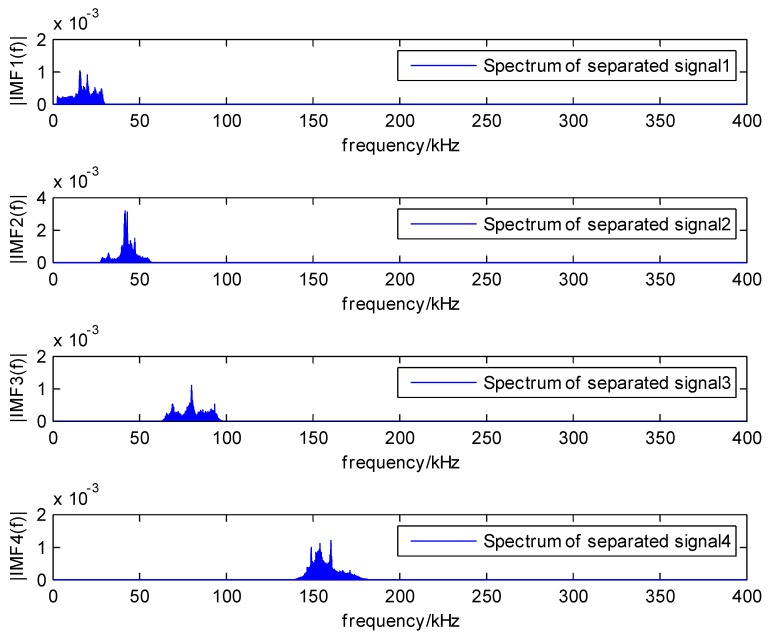
The spectrum of separated signals of sensor 1.

**Figure 19:**
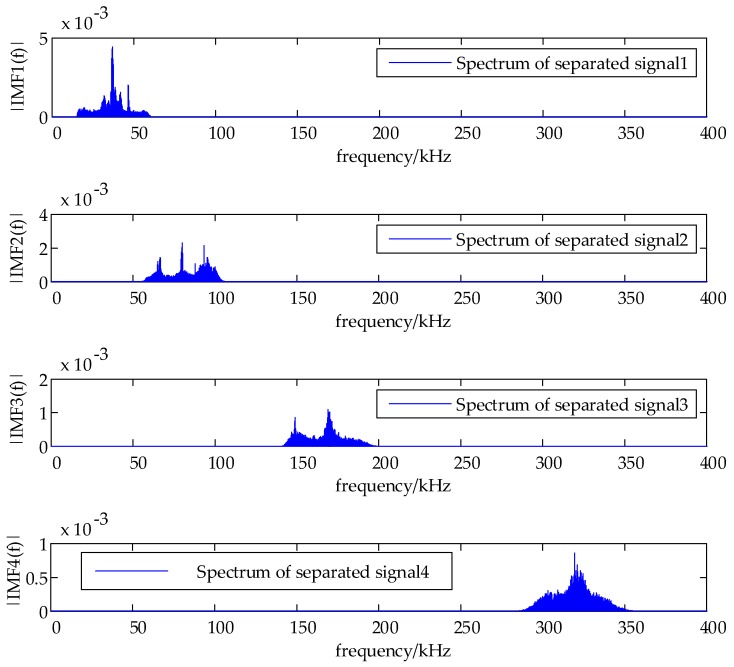
The spectrum of separated signals of sensor 2.

The load speed is 5 mm/min, AE signals happen on the load force of 200 N, and the plate is broken when the load force is about 220 N. The spectrum and local maxima by the proposed method are shown in Figure 17. In addition, the spectrum of separated signals from two sensors are depicted in Figure 18 and Figure 19.

## 5. Conclusions

This paper presents a novel signal decomposition method which is applied to AE signal processing. To eliminate the impact of noise interference or frequency dispersion, the LWM segmentation approach is used to search the dominant local maxima and the segmentation boundaries. Through the presented algorithm, useful components for damage analysis are decomposed. The decomposition results from the simulation and practical experiment show that the meaningful empirical modes, which are more reflective of damage characteristics, are obtained. Additionally, compared with the original EWT, components decomposed by the proposed LWM-EWT are much more reasonable for the AE signals. Since the frequency of AE signals can be influenced by different structures, further research work should concentrate on acquiring more signals from other structures, to validate the proposed method.

## Figures and Tables

**Table 1:** The detailed information of the experimental system.

Name	Type	Parameter
CFRP plate with stiffeners	TR50S12L	650 mm × 650 mm × 3 mm, 20 layers, [0, 90]_10S_; T shape stiffeners (cross section): 40 mm × 20 mm × 3 mm
Pencil-lead	Mitsu Nano Dia	Diameter: 0.5 mm
AE sensor	PAC ^1^ WS α	Wide-band (WD) Sensor; Operating Frequency Range: 100–1000 kHz; Temperature Range: −65–175 °C.
Pre-amplifier	PAC 2/4/6	Gain Selectable: 20/40/60 dB Bandwidth: 10 kHz–2.5 MHz (20 dB), 10 kHz–2.0 MHz (40 dB), 10 kHz–900 kHz (60 dB); Temperature Range: −40–65 °C
AE system	PAC PCI-2	2 simultaneous channels, 18 bit resolution, 40 MS/s
Industrial computer	IEI RACK-360GBATX	E5800 3.2 GHz, 4G memory, 7 PCI slots

^1^ Physical Acoustics Corporation.
